# Surgical excision and radiotherapy for brain metastasis from colorectal cancer: How frailty and comorbidity indices influence outcome

**DOI:** 10.1002/kjm2.12815

**Published:** 2024-03-14

**Authors:** Chia‐En Wong, Yu Chang, Chi‐Chen Huang, Hao‐Hsiang Hsu, Yu‐Hsuan Lai, Kwang‐Yu Chang, Chih‐Yuan Huang, Liang‐Chao Wang, Jung‐Shun Lee, Po‐Hsuan Lee

**Affiliations:** ^1^ Division of Neurosurgery, Department of Surgery National Cheng Kung University Hospital, College of Medicine, National Cheng Kung University Tainan Taiwan; ^2^ Department of Oncology National Cheng Kung University Hospital, College of Medicine, National Cheng Kung University Tainan Taiwan; ^3^ Department of Radiation Oncology National Cheng Kung University Hospital, College of Medicine, National Cheng Kung University Tainan Taiwan; ^4^ National Institute of Cancer Research, National Health Research Institutes Tainan Taiwan; ^5^ Department of Cell Biology and Anatomy College of Medicine, National Cheng Kung University Tainan Taiwan; ^6^ Institute of Basic Medical Sciences, College of Medicine, National Cheng Kung University Tainan Taiwan

**Keywords:** brain metastases, colorectal cancer, comorbidity index, modified frailty index, prognostic nutritional index

## Abstract

The incidence of brain metastasis (BM) from colorectal cancer (CRC) is increasing. This study aims to identify the clinical prognosticators and evaluate the prognostic validity of common comorbidity indices in patients with BM from CRC. This retrospective single‐center study analyzed 93 patients with BM from CRC who received surgical excision and/or radiotherapy. The clinical characteristics and prognostic indices including the 5‐item modified frailty index (mFI‐5) and prognostic nutritional index (PNI) were calculated from the collected patient data and analyzed. In this study, 66 (71.0%), 10 (10.8%), and 17 (18.3%) patients received whole‐brain radiotherapy (WBRT) alone, surgery alone, and surgery plus WBRT, respectively. The median survival of all patients was 3.98 months (IQR: 1.74–7.99). The 2‐ and 3‐year survival rates were 7.4% and 3.7%, respectively. Controlled primary tumor (*p* = 0.048), solitary BM (*p* = 0.001), surgery + radiation (*p* < 0.001), and greater PNI (*p* = 0.001) were independent predictors of favorable survival. In surgically treated patients, uncontrolled primary tumor (*p* = 0.006), presence of multiple BM (*p* < 0.001), and MFI‐5 ≥ 2 (*p* = 0.038) were independent prognosticators. For patients who received WBRT, the presence of two (*p* = 0.004) or multiple (*p* < 0.001) BM and PNI (*p* < 0.001) were independent survival predictors MFI‐5, multiple BM, and the status of the primary tumor were independent prognosticators for patients who underwent surgery for CRCBM. For patients who received WBRT, the PNI and the number of BM were independent survival predictors.

## 1 INTRODUCTION

Brain metastasis (BM) from colorectal cancer (CRC) is an emerging challenge for neurosurgeons and neuro‐oncologists because of the advances in therapeutic modalities and improved survival in patients with CRC.^1^ The incidence of BM in patients with CRC was reported to be up to 13%,^1^, ^2^ and most patients had BM detected more than 1 year after the diagnosis of metastatic CRC.^3^ Most patients with BM from CRC have unfavorable survival outcomes despite aggressive treatment strategies, including surgical excision, radiotherapy, radiosurgery, and systemic therapy.^3^, ^4^ The reported median survival time after the diagnosis of BM varies from 2.5 to 87 months.^5^, ^6^ In recent studies, surgical excision of resectable colorectal cancer brain metastasis (CRCBM) has been increasingly reported to improve overall survival (OS).^7^, ^8^, ^9^, ^10^ Moreover, the survival of patients with all BM has been shown to be related to several patient characteristics, including age, presence of extracranial metastases, being underweight, and medical comorbidities.^10^, ^11^, ^12^, ^13^

Comorbidity indices, such as the Recursive Partitioning Analysis (RPA) class, the 5‐item modified frailty index (MFI‐5) and prognostic nutritional index (PNI), have been increasingly utilized in both oncological and neurosurgical practices to represent a comprehensive comorbidity status that aims to improve the prediction of prognosis.^14^, ^15^, ^16^, ^17^, ^18^ For instance, a higher MFI‐5 score predicted worse early outcomes and 30‐day readmissions following CRC surgeries.^19^, ^20^ The PNI has been shown to be a prognostic indicator for survival in patients with BM from non‐small cell lung cancer and glioblastoma.^21^, ^22^ However, despite the growing literature supporting the validity of these comorbidity scores, studies specifically evaluating the comorbidity indices for predicting survival outcomes in patients with BM from CRC are lacking, and the prognostic values of these comorbidity indices in CRCBM patients remain unclear.

To address the clinical knowledge gap, this study aimed to identify the clinical prognosticators and evaluate the prognostic validity of five comorbidity indices in patients with BM from CRC in a single‐center retrospective analysis. The knowledge gained in the present study may help clinicians to better identify high‐risk patients who may benefit from additional clinical attention.

## 2 METHODS

The present study is a retrospective single‐center analysis of patients with BM from CRC. This study was approved by the institutional review board. Considering the retrospective design of the study, the requirement for informed consent was waived.

Patients with histologically proven colorectal adenocarcinoma and a histological or radiological diagnosis of BM between January 2010 and December 2021 were included. Patients with non‐adenocarcinoma CRC, leptomeningeal carcinomatosis, or secondary malignancies were excluded. All patients had at least 6 months of follow‐up. The OS after BM was calculated from the date of radiographic documentation of BM to the date of the patient's death or the last follow‐up investigation. Data for all patients were compiled from electronic medical records and clinical notes.

Primary treatments for CRC BM were categorized as the following: 1. Patients who underwent surgery and subsequent postoperative whole‐brain radiotherapy (WBRT) were assigned as surgery + WBRT group. 2. Patients who received surgery alone without postoperative WBRT were assigned as surgery group. 3. Patients who received WBRT alone assigned as WBRT group. Steroids were prescribed according to patients' symptom in all groups. Patients who did not received either surgery or WBRT for BM were excluded.

Prognostic indices, including the MFI‐5, PNI, systemic immune‐inflammation index (SIII), neutrophil‐to‐lymphocyte ratio (NLR), and platelet‐to‐lymphocyte ratio (PLR) were calculated from the collected patient data. The extent of brain metastasis was evaluated using gadolinium‐enhanced magnetic resonance imaging (MRI).

Categorical variables are presented as numbers and percentages. Continuous variables are presented as mean ± standard deviation for parametric values, and median and interquartile range (IQR) for non‐parametric values. Comparisons of categorical and continuous variables were performed using the *χ* ^2^ test and analysis of variance (ANOVA), respectively. Kaplan–Meier survival analysis was used to analyze OS, and the difference between the groups was calculated using the log‐rank test. The Cox proportional hazards method was used to create the regression model and estimate hazard ratios. Variables with a *p*‐value of <0.2 in the univariate Cox analysis were selected for further multivariate analysis of OS. Multicollinearity was checked, and none of the variables in the final regression model exhibited a variance inflation factor greater than 2. Statistical tests were conducted using MedCalc 19.7.2 (MedCalc Software, Ltd.). Statistical significance was set at *p* < 0.05.

## 3 RESULTS

### 3.1 Demographics

A total of 93 patients were included in this study. The mean age of the patients was 63.1 ± 10.9, with 46 (49.5%) patients being male. Regarding the primary tumor, 57 (61.3%) patients had a rectosigmoid origin, and the primary CRC was under control in 37 (39.8) patients. KRAS mutation status was examined in 57 (61.3%) patients, among whom 25 (26.9%) and 32 (34.4%) had wild type and mutant KRAS, respectively. Prior to the diagnosis of BM, 79 (84.9%) patients received at least one cycle of chemotherapy. Comparing patients with different BM treatment, patients who received surgical treatment had lower MFI‐5 score (*p* = 0.031) and had higher percentage of postoperative chemotherapy (*p* = 0.015) (Table 1).

**TABLE 1:** Patient characteristics.

Clinical characteristics	Overall, *N* = 93	Surgery, *N* = 10	Surgery + WBRT, *N* = 17	WBRT, *N* = 66	*p*‐Value
Demographics					
Age	63.1 ± 10.9	65.3 ± 11.5	58.6 ± 10.3	63.9 ± 10.8	0.159
Sex: male	46 (49.5)	5 (50.0)	11 (64.7)	30 (45.5)	0.367
Overall survival (months)	3.98 (1.74–7.99)	3.80 (3.26–9.63)	11.73 (7.32–19.96)	2.89 (1.45–5.10)	**<0.001**
Primary tumor status					
Primary tumor origin					
Colon	36 (38.7)	7 (70.0)	10 (58.8)	39 (59.1)	0.799
Rectosigmoid	57 (61.3)	3 (30.0)	7 (41.2)	27 (40.9)	0.799
Primary tumor controlled	37 (39.8)	4 (40.0)	11 (64.7)	22 (33.3)	0.062
Extracranial metastases	87 (93.5)	9 (90.0)	16 (94.1)	62 (93.9)	0.889
KRAS					
Wild type	25 (26.9)	2 (20.0)	4 (23.5)	19 (28.8)	0.168
Mutant	32 (34.4)	3 (30.0)	10 (58.8)	19 (28.8)	0.168
Unknown	36 (38.7)	5 (50.0)	3 (17.7)	28 (42.4)	0.168
Brain metastases status					
Number of BM					
1	52 (55.9)	7 (70.0)	13 (76.4)	32 (48.5)	0.196
2	11 (11.8)	1 (10.0)	2 (11.8)	8 (12.1)	
≥ 3	30 (32.3)	2 (20.0)	2 (11.8)	26 (39.4)	
Infratentorial involvement	51 (54.8)				
Systemic treatment					
Received chemotherapy before BM	79 (84.9)	8 (80.0)	13 (76.4)	58 (87.9)	0.452
Received chemotherapy after BM	49 (52.7)	9 (90.0)	11 (64.7)	29 (43.9)	**0.015**
Prognostic indices prior to BM treatment					
Modified frailty index					**0.031**
0	35 (37.6)	6 (60.0)	11 (64.7)	18 (27.3)	
1	33 (35.5)	2 (20.0)	4 (23.5)	27 (40.9)	
≥ 2	25 (26.9)	2 (20.0)	2 (11.8)	21 (31.8)	
Prognostic nutritional index	41.9 (36.6–46.7)	41.9 (36.9–45.0)	43.0 (39.2–47.9)	40.5 (36.0–46.5)	0.574
Systemic immune‐inflammation index	1404 (691–2983)	2525 (1156–3700)	1940 (852–4259)	1323 (593–2592)	0.110
Neutrophil‐to‐lymphocyte ratio	7.5 (3.8–13.4)	13.3 (6.6–21.7)	8.5 (3.5–13.4)	6.3 (3.4–12.1)	0.066
Platelet‐to‐lymphocyte ratio	323 (130–634)	162 (63–440)	311 (147–716)	352 (130–708)	0.408

*Note*: Bold values indicate *p* < 0.05.

### 3.2 The status of BM at diagnosis

Among the 93 patients, 52, 11, and 30 patients had single, two, and multiple (≥3) BM detected by gadolinium‐enhanced MRI at the time of BM diagnosis, respectively. Infratentorial involvement was observed in 51 (54.8%) patients. For the treatment of BM, 66 (71.0%), 10 (10.8%), and 17 (18.3%) patients received WBRT alone, surgery alone, and surgery plus WBRT, respectively. Fifty‐one (54.8%) patients received at least one cycle of chemotherapy after diagnosis of BM. The value of the prognostic indices calculated from the last available clinical and laboratory data prior to BM treatment are shown in Table 1.

### 3.3 Survival outcomes

**FIGURE 1:**
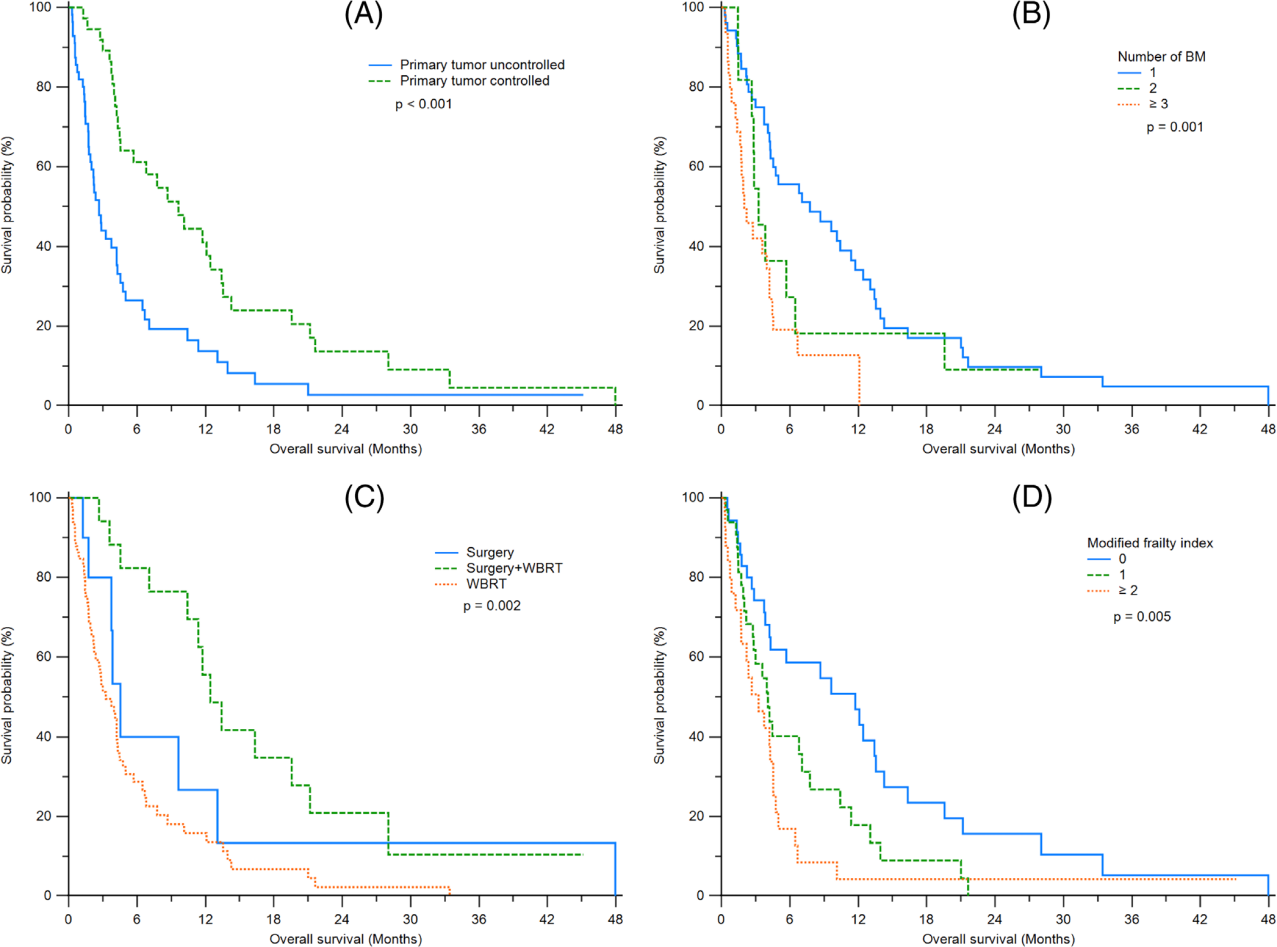
Kaplan–Meier survival analysis curve for OS. The survival curves between patients with controlled and uncontrolled primary CRC (A), different number of BM (B), treatment of BM (C), and MFI‐5 scores (D).

The median OS of all patients was 3.98 months (IQR: 1.74–7.99). The 2‐ and 3‐year survival rates were 7.4% and 3.7%, respectively. Patients who received surgery + WBRT had significantly longer OS compared to patients who received surgery alone and WBRT alone (*p* < 0.001) (Table 1). Patients with controlled primary tumors had better survival outcomes compared to those with uncontrolled primary CRC (9.63 vs. 2.66 months; *p* < 0.001) (Figure 1A). An increased number of BM was associated with worse outcomes (7.66 vs. 3.19 vs. 2.01 months; *p* = 0.001) (Figure 1B). The survival outcomes of each BM treatment group are shown in Figure 1C. Statistically significant difference was seen among the three groups (3.25 vs. 4.47 vs. 12.10 months for WBRT, surgery, and surgery + WBRT, respectively; *p* = 0.002). A greater MFI‐5 score was correlated to worse survival (11.38 vs. 4.08 vs. 3.19 months; *p* = 0.005) (Figure 1D).

### 3.4 Survival prognosticators

Univariate Cox regression showed that patients with multiple BM and greater MFI‐5 scores were associated with worse survival, whereas patients with controlled primary tumors, who received surgery + WBRT, and those with a greater PNI were associated with better survival (Table 2). Multivariate Cox regression revealed that a controlled primary tumor (*p* = 0.048), multiple BM (*p* = 0.001), receiving surgery + WBRT for BM (*p* < 0.001), and greater PNI (*p* = 0.001) were independent factors that predicted survival (Table 2). The RPA class, SIII, NLR, and PLR were not significantly associated with OS.

**TABLE 2:** Cox regression model of overall survival.

Characteristics	Univariate Cox regression			Multivariate Cox regression		
	OR	95% CI	*p*‐Value	OR	95% CI	*p*‐Value
Pre‐treatment demographics						
Controlled primary tumor	0.416	0.261–0.664	**<0.001**	0.578	0.333–0.997	**0.048**
Extracranial metastases	1.110	0.446–2.761	0.832			
Number of BM						
1	Reference			Reference		
2	1.409	0.700–2.836	0.290	1.508	0.705–3.229	0.290
≥3	2.487	1.345–4.601	**<0.001**	2.694	1.504–4.825	**0.001**
Infratentorial involvement	1.009	0.643–1.584	0.968			
KRAS: mutant	0.652	0.349–1.218	0.179	1.414	0.795–2.515	0.239
Age	1.017	0.995–1.039	0.127	1.001	0.974–1.029	0.928
Sex: male	0.656	0.415–1.036	0.101	0.657	0.397–1.087	0.112
Rectosigmoid origin	1.167	0.733–1.857	0.516			
Received chemotherapy before BM	0.855	0.463–1.581	0.617			
Treatment strategies						
Treatment for BM						
WBRT	Reference			Reference		
Surgery	0.578	0.290–1.151	0.130	0.540	0.233–1.250	0.150
Surgery + WBRT	0.356	0.219–0.580	**<0.001**	0.232	0.107–0.504	**<0.001**
Received chemotherapy after BM	0.660	0.416–1.046	0.077	0.698	0.421–1.160	0.166
Prognostic indices prior to BM treatment						
Modified frailty index						
0	Reference			Reference		
1	1.719	1.031–2.864	**0.034**	0.978	0.531–1.802	0.943
≥2	2.397	1.308–4.395	**0.001**	1.500	0.870–3.358	0.124
Prognostic nutritional index	0.967	0.940–0.995	**0.020**	0.942	0.909–0.977	**0.001**
Systemic immune‐inflammation index	1.000	1.000–1.001	0.469			
Neutrophil‐to‐lymphocyte ratio	1.012	0.988–1.037	0.315			
Platelet‐to‐lymphocyte ratio	1.000	0.999–1.001	0.455			

*Note*: Bold values indicate *p* < 0.05.

### 3.5 Subgroup analyses for surgically treated and non‐surgically treated patients

Subgroup analyses were performed for patients who underwent surgery (surgery and surgery + WBRT) or radiation therapy (WBRT) as the primary treatment for BM. For surgically treated patients, uncontrolled primary tumor (*p* = 0.006), presence of multiple BM (*p* < 0.001), and MFI‐5 ≥ 2 (*p* = 0.038) were independent poor prognosticators of OS (Table 3). For patients who received WBRT, the presence of two (*p* = 0.004) or multiple (*p* < 0.001) BM and PNI (*p* < 0.001) were independent predictors of OS (Table 4).

**TABLE 3:** Subgroup analysis of patients receiving surgical excision of BM.

Characteristics	Univariate Cox regression			Multivariate Cox regression		
	OR	95% CI	*p*‐Value	OR	95% CI	*p*‐Value
Pre‐treatment demographics						
Controlled primary tumor	0.463	0.180–1.188	0.109	0.063	0.009–0.553	**0.006**
Extracranial metastases	3.821	0.822–17.735	0.088			
Number of BM						
1	Reference			Reference		
2	0.667	0.148–3.006	0.290	0.755	0.091–6.256	0.427
≥ 3	12.054	2.896–25.167	**0.001**	7.678	1.504–37.809	**< 0.001**
Infratentorial involvement	1.036	0.426–2.518	0.939			
KRAS: mutant	1.234	0.339–4.484	0.750			
Age	1.017	0.995–1.039	0.127	1.020	0.960–1.084	0.524
Sex: male	0.920	0.368–2.304	0.859			
Rectosigmoid origin	1.291	0.527–3.165	0.577			
Received chemotherapy before BM	0.901	0.350–2.323	0.829			
Treatment strategies						
Treatment for BM						
Surgery	Reference			Reference		
Surgery + WBRT	0.486	0.168–1.408	0.184	0.220	0.046–1.059	0.059
Received chemotherapy after BM	0.818	0.310–2.158	0.684			
Prognostic indices prior to BM treatment						
Modified frailty index						
0	Reference			Reference		
1	3.109	1.058–8.130	**0.039**	1.028	0.907–1.487	0.095
≥2	3.453	1.097–5.305	**0.032**	2.247	1.194–4.167	**0.038**
Prognostic nutritional index	0.972	0.916–1.029	0.325	1.036	0.922–1.166	0.547
Systemic immune‐inflammation index	0.999	0.999–1.000	0.224	0.999	0.999–1.000	0.325
Neutrophil‐to‐lymphocyte ratio	0.980	0.925–1.039	0.501			
Platelet‐to‐lymphocyte ratio	1.000	0.999–1.001	0.898			

*Note*: Bold values indicate *p* < 0.05.

**TABLE 4:** Subgroup analysis of patients receiving radiotherapy for BM.

Characteristics	Univariate Cox regression			Multivariate Cox regression		
	OR	95% CI	*p*‐Value	OR	95% CI	*p*‐Value
Pre‐treatment demographics						
Controlled primary tumor	0.401	0.224–0.717	**0.002**	0.715	0.364–1.405	0.331
Extracranial metastases	1.116	0.385–2.976	0.895			
Number of BM						
1	Reference			Reference		
2	2.029	0.886–4.643	0.094	4.511	1.622–12.547	**0.004**
≥3	1.865	1.010–3.443	**0.046**	4.260	1.989–9.122	**<0.001**
Infratentorial involvement	0.983	0.580–1.665	0.948			
KRAS: mutant	0.655	0.322–1.322	0.356			
Age	1.014	0.988–1.040	0.304			
Sex: male	0.596	0.349–1.017	0.058	0.616	0.376–1.026	0.068
Rectosigmoid origin	0.995	0.570–1.749	0.996			
Received chemotherapy before BM	0.313	0.137–0.916	0.035	0.767	0.299–1.971	0.582
Treatment strategies						
Received chemotherapy after BM	0.828	0.485–1.413	0.489			
Prognostic indices prior to BM treatment						
Modified frailty index						
0	Reference			Reference		
1	1.201	0.616–2.341	**0.590**	0.973	0.469–2.017	0.941
≥2	2.158	1.075–4.329	**0.030**	1.758	0.795–3.884	0.163
Prognostic nutritional index	0.891	0.851–0.933	**<0.001**	0.865	0.817–0.916	**<0.001**
Systemic immune‐inflammation index	1.000	1.000–1.001	0.036	1.001	0.999–1.001	0.214
Neutrophil‐to‐lymphocyte ratio	1.041	1.016–1.066	0.004	0.984	0.898–1.078	0.728
Platelet‐to‐lymphocyte ratio	1.000	0.999–1.001	0.039	1.000	0.999–1.001	0.319

*Note*: Bold values indicate *p* < 0.05.

## 4 DISCUSSION

Patients with BM from CRC have heterogeneous characteristics and their management strategies can be complex. Optimal risk stratification strategies for these patients are limited. The present study evaluated the prognostic validity of five comorbidity indices in patients with BM from CRC. Our results showed that MFI‐5, along with the presence of multiple BM and the status of the primary tumor, were independent prognosticators for patients who underwent surgical resection of CRCBM. Whereas for patients who received WBRT for CRCBM, PNI and the number of BM were independent predictors of survival.

Patients with BM represent a diverse population.^23^ In state‐of‐the‐art practice, BM are considered a special site of metastatic cancer rather than a single disease entity.^24^ In this study, we specifically focused on BM from CRC, since it is an emerging condition due to the advances in therapeutic modalities and improved survival in patients with CRC. Given the diverse patient characteristics, risk stratification for these patients is pivotal and can provide valuable information to facilitate the identification of high‐risk patients who may benefit from additional clinical attention and help clinicians in patient counseling and making decisions regarding treatment. Therefore, we aimed to identify patient, CRC, BM, and comorbidity‐related factors that could predict patient outcomes.

We investigated the validity of five prognostic indices, including the MFI‐5, PNI, SIII, NLR, and PLR in patients with BM from CRC, and our results demonstrated that a greater PNI was an independent predictor of favorable survival. PNI is a nutrition‐immune parameter based on serum albumin level and lymphocyte count.^17^ Hypoalbuminemia can result in dysregulated immune function, abnormal activation of systemic inflammation, and loss of circulating drug efficacy.^25^ Albumin binds to endogenous ligands and the albumin‐drug complex serves as a drug reservoir that can enhance drug biodistribution and bioavailability. Therefore, hypoalbuminemia is reported to be associated with increased drug clearance, cancer cachexia, and elevated protein turnover secondary to chronic inflammation.^25^, ^26^, ^27^ Consequently, it is reasonable that a low albumin level could favor tumor development and cancer‐related inflammation, which worsens outcome.^25^, ^26^, ^27^ Likewise, lymphocytes are involved in anti‐tumoral immunity, and lymphocyte dysfunction or imbalance is associated with cancer progression.^28^ Previous studies have shown that pretreatment PNI predicted the outcome in patients with CRC,^29^, ^30^ and our results further validated the prognostic value of PNI in patients with BM from CRC. Overall, both malnutrition and immune dysfunction represent unfavorable conditions in the prognosis of patients with cancer.

Besides the comorbidity indices, our analyses also revealed that the control of the primary CRC and the presence of multiple BM were independent factors that predicted survival. The impact of coexisting extracranial metastases (ECM) and the number of BM at the time of the diagnosis of BM have been reported and were shown to be associated with worse survival.^1^, ^8^, ^10^, ^23^, ^31^, ^32^ Thurmaier and colleagues reported that the pattern of ECM and the control of primary tumor were also relevant to survival, with patients with concurrent liver and lung metastasis demonstrating the worst outcomes.^23^ In our results, although ECM was not associated with survival, we showed that patients who had controlled primary tumor had better survival. This finding might suggest that for CRC patients with BM and ECM, as long as the primary tumor and ECM were controlled, resection of BM could potentially improve survival. Additionally, the association between the number of BM detected by MRI at the initial diagnosis and outcomes has also been described in the literature.^8^, ^10^, ^31^, ^32^

It should be noted that the presence of multiple metastases may influence the decision‐making regarding local treatment for BM, although multiple BM are not a contraindication for metastasectomy.^33^ The primary treatment modality selected for BM was significantly associated with patient outcome. Several studies have demonstrated the survival benefits of surgical resection of BM from CRC in selected patients.^3^, ^7^, ^8^, ^9^, ^10^ Likewise, our results also showed that patients who underwent surgery + WBRT survived longer compared with those treated non‐operatively. These findings further demonstrated that patients with BM indeed represent a heterogeneous population, and the effect of potential treatment allocation bias could not be clearly assessed if they were evaluated together.^23^

To minimize the bias in patient selection and the survival difference between surgery and WBRT, and to further evaluate the validity of the comorbidity indices in each patient population, we performed subgroup analyses in patients who received surgery and WBRT as primary treatment for BM. In the surgery subgroup, MFI‐5, but not PNI, was an independent prognosticator of OS. In the overall analysis, there was indeed an association between a higher MFI‐5 score and worse prognosis in the univariate analysis and a similar trend in the multivariate analysis. A probable explanation for this discrepancy is that for patients who underwent surgery, frailty and medical comorbidities may have a greater impact on the perioperative outcomes. Frailty status has been reported to predict postoperative complications, length of hospitalization, and discharge disposition in patients undergoing brain tumor surgery.^34^, ^35^, ^36^ Moreover, associations between patient frailty and survival outcomes after surgery for BM have also been reported in the literature.^36^, ^37^

In the WBRT subgroup, the results were similar to that of the overall analysis, with the number of BM and PNI shown to be independent survival prognosticators. Nonetheless, compared with the overall analysis, there was a slight difference in the WBRT subgroup results, which is the effect of the number of BM. In the overall analysis, only the presence of multiple (≥3) BM was associated with worse survival, whereas in the WBRT subgroup, both two and multiple BM were associated with worse survival. Given that patients with one or two BM may be more likely to receive surgical resection and thereby have a better outcome compared with those with multiple BM, it is probable that the survival difference between patients with one or two BM might be less in the surgical or overall cohort. In contrast, in the non‐surgical subgroup, since an increasing number of BM represents a more aggressive disease status, the number of BM may be more likely to have a dose‐dependent effect on survival.^8^, ^10^, ^23^, ^31^

The findings of the present study should be interpreted in light of some limitations. First, this study was a single‐center study with a limited number of patients. Second, although we reported the KRAS mutation status in the majority of our patients, BRAF V600E mutation status was only available in a small group of patients and the number of these patients was insufficient for analysis. Future studies are required to evaluate the impact of these genes potentially relevant to the survival of CRC BM patients. Third, although surgery and WBRT were compared, the effect of stereotactic radiosurgery (SRS) was not evaluated due to unavailability of SRS in our institution. Furthermore, the clonal evolution of metastatic CRC was reported and might have an impact on patient outcomes, but this information was unavailable in our patients because most patients who had a recurrence of BM did not receive a second tissue proof. Since stereotactic radiosurgery was not yet a treatment modality for CRC brain metastasis at our institute, the outcome remains to be investigated.

## 5 CONCLUSIONS

In conclusion, this study evaluated the prognostic validity of five comorbidity indices in patients with BM from CRC. Our results showed that MFI‐5, along with the presence of multiple BM and the status of the primary tumor, were independent prognosticators for patients who underwent surgical resection of CRCBM. For patients who received WBRT for CRCBM, the PNI and the number of BM were independent predictors of survival. The knowledge gained in the present study may provide valuable information for the identification of high‐risk patients who may benefit from additional clinical attention and guide clinicians in patient counseling and decision‐making regarding treatment.

## CONFLICT OF INTEREST STATEMENT

The authors declare no conflicts of interest related to this paper.

## ETHICAL APPROVAL

The study was reviewed and approved by the Institutional Review Board (NCKU‐IRB‐Approval No. B‐ER‐111‐370).

## INFORMED CONSENT STATEMENT

Patient consent was waived due to the retrospective nature of the study, as approved by the Institutional Review Board.

## References

[1] Nieder C , Spanne O , Mehta MP , Grosu AL , Geinitz H . Presentation, patterns of care, and survival in patients with brain metastases. Cancer. 2011;117:2505–2512. 24048799 10.1002/cncr.25707

[2] Christensen TD , Spindler K‐LG , Palshof JA , Nielsen DL . Systematic review: brain metastases from colorectal cancer—incidence and patient characteristics. BMC Cancer. 2016;16:260. 27037031 10.1186/s12885-016-2290-5 PMC4818396

[3] Baek JY , Kang MH , Hong YS , Kim TW , Kim DY , Oh JH , et al. Characteristics and prognosis of patients with colorectal cancer‐associated brain metastases in the era of modern systemic chemotherapy. J Neurooncol. 2011;104:745–753. 21336772 10.1007/s11060-011-0539-z

[4] Byrne BE , Geddes T , Welsh FKS , John TG , Chandrakumaran K , Rees M . The incidence and outcome of brain metastases after liver resection for colorectal cancer metastases. Colorectal Dis. 2012;14:721–726. 21834877 10.1111/j.1463-1318.2011.02762.x

[5] Farnell GF , Buckner JC , Cascino TL , O'Connell MJ , Schomberg PJ , Suman V . Brain metastases from colorectal carcinoma: the long term survivors. Cancer. 1996;78:711–716. 8756361 10.1002/(SICI)1097-0142(19960815)78:4<711::AID-CNCR3>3.0.CO;2-H

[6] Ko FC , Liu JM , Chen WS , Chiang JK , Lin TC , Lin JK . Risk and patterns of brain metastases in colorectal cancer. Dis Colon Rectum. 1999;42:1467–1471. 10566536 10.1007/BF02235049

[7] Damiens K , Ayoub JPM , Lemieux B , Aubin F , Saliba W , Campeau MP , et al. Clinical features and course of brain metastases in colorectal cancer: an experience from a single institution. Curr Oncol. 2012;19:254–258. 23144573 10.3747/co.19.1048 PMC3457876

[8] Suzuki Y , Yamaguchi T , Matsumoto H , Nakano D , Honda G , Shinoura N , et al. Prognostic factors and treatment effects in patients with curatively resected brain metastasis from colorectal cancer. Dis Colon Rectum. 2014;57:56–63. 24316946 10.1097/01.dcr.0000436998.30504.98

[9] Rico GT , Price TJ , Karapetis C , Piantadosi C , Padbury R , Roy A , et al. Brain metastasis in advanced colorectal cancer: results from the South Australian metastatic colorectal cancer (SAmCRC) registry. Cancer Biol Med. 2017;14:371–376. 29372103 10.20892/j.issn.2095-3941.2017.0068 PMC5785167

[10] Chang Y , Wong CE , Lee PH , Huang CC , Lee JS . Survival outcome of surgical resection vs. radiotherapy in brain metastasis from colorectal cancer: a meta‐analysis. Front Med (Lausanne). 2022;9:9. 10.3389/fmed.2022.768896 PMC8957984 35350580

[11] Ning WZ , Bing JX , Lu J , Yu GX , Qiang HZ , Duan H , et al. Survival benefit from surgical resection in lung cancer patients with brain metastases: a single‐center, propensity‐matched analysis cohort study. Ann Surg Oncol. 2022;29:3684–3693. 35181815 10.1245/s10434-022-11365-y

[12] Lareida A , Terziev R , Grossenbacher B , Andratschke N , Roth P , Rohrmann S , et al. Underweight and weight loss are predictors of poor outcome in patients with brain metastasis. J Neurooncol. 2019;145:339–347. 31571112 10.1007/s11060-019-03300-1

[13] Schneider M , Heimann M , Schaub C , Eichhorn L , Potthoff AL , Giordano FA , et al. Comorbidity burden and presence of multiple intracranial lesions are associated with adverse events after surgical treatment of patients with brain metastases. Cancers (Basel). 2020;12:1–8. 10.3390/cancers12113209 PMC7692304 33142701

[14] Dicpinigaitis AJ , Kalakoti P , Schmidt M , Gurgel R , Cole C , Carlson A , et al. Associations of baseline frailty status and age with outcomes in patients undergoing vestibular schwannoma resection. JAMA Otolaryngol–Head Neck Surg. 2021;147:608–614. 33914061 10.1001/jamaoto.2021.0670 PMC8085763

[15] Tang OY , Bajaj AI , Zhao K , Liu JK . Patient frailty association with cerebral arteriovenous malformation microsurgical outcomes and development of custom risk stratification score: an analysis of 16,721 nationwide admissions. Neurosurg Focus. 2022;53:E14. 10.3171/2022.4.FOCUS2285 35901730

[16] Khalafallah AM , Huq S , Jimenez AE , Brem H , Mukherjee D . The 5‐factor modified frailty index: an effective predictor of mortality in brain tumor patients. J Neurosurg. 2020;135:78–86. 32796147 10.3171/2020.5.JNS20766

[17] Ni L , Huang J , Ding J , Kou J , Shao T , Li J , et al. Prognostic nutritional index predicts response and prognosis in cancer patients treated with immune checkpoint inhibitors: a systematic review and meta‐analysis. Front Nutr. 2022;9:9. 10.3389/fnut.2022.823087 PMC9353139 35938131

[18] Xu YS , Liu G , Zhao C , Lu SL , Long CY , Zhong HG , et al. Prognostic value of combined preoperative carcinoembryonic antigen and prognostic nutritional index in patients with stage II–III colon cancer. Front Surg. 2021;8:8. 10.3389/fsurg.2021.667154 PMC8329091 34355011

[19] Tatar C , Benlice C , Delaney CP , Holubar SD , Liska D , Steele SR , et al. Modified frailty index predicts high‐risk patients for readmission after colorectal surgery for cancer. Am J Surg. 2020;220:187–190. 31735257 10.1016/j.amjsurg.2019.11.016

[20] AL‐Khamis A , Warner C , Park J , Marecik S , Davis N , Mellgren A , et al. Modified frailty index predicts early outcomes after colorectal surgery: an ACS‐NSQIP study. Colorectal Dis. 2019;21:1192–1205. 31162882 10.1111/codi.14725

[21] Li W , Qu Y , Wen F , Yu R , He X , Jia H , et al. Prognostic nutritional index and systemic immune‐inflammation index are prognostic biomarkers for non‐small‐cell lung cancer brain metastases. Biomark Med. 2021;15:1071–1084. 34397267 10.2217/bmm-2020-0786

[22] Huq S , Khalafallah AM , Botros D , Oliveira LAP , White T , Dux H , et al. The prognostic impact of nutritional status on postoperative outcomes in glioblastoma. World Neurosurg. 2021;146:e865–e875. 33197633 10.1016/j.wneu.2020.11.033

[23] Thurmaier J , Heinemann V , Engel J , Schubert‐Fritschle G , Wiedemann M , Nüssler NC , et al. Patients with colorectal cancer and brain metastasis: the relevance of extracranial metastatic patterns predicting time intervals to first occurrence of intracranial metastasis and survival. Int J Cancer. 2021;148:1919–1927. 33113215 10.1002/ijc.33364

[24] Mitsuya K , Nakasu Y , Kurakane T , Hayashi N , Harada H , Nozaki K . Elevated preoperative neutrophil‐to‐lymphocyte ratio as a predictor of worse survival after resection in patients with brain metastasis. J Neurosurg. 2017;127:433–437. 27911233 10.3171/2016.8.JNS16899

[25] Moujaess E , Fakhoury M , Assi T , Elias H , el Karak F , Ghosn M , et al. The therapeutic use of human albumin in cancer patients' management. Crit Rev Oncol Hematol. 2017;120:203–209. 29198333 10.1016/j.critrevonc.2017.11.008

[26] Turner DC , Kondic AG , Anderson KM , Robinson AG , Garon EB , Riess JW , et al. Pembrolizumab exposure‐response assessments challenged by Association of Cancer Cachexia and Catabolic Clearance. Clin Cancer Res. 2018;24:5841–5849. 29891725 10.1158/1078-0432.CCR-18-0415

[27] Larsen MT , Kuhlmann M , Hvam ML , Howard KA . Albumin‐based drug delivery: harnessing nature to cure disease. Mol Cell Ther. 2016;4:4. 10.1186/s40591-016-0048-8 PMC4769556 26925240

[28] Gupta D , Lis CG . Pretreatment serum albumin as a predictor of cancer survival: a systematic review of the epidemiological literature. Nutr J. 2010;9:1–16. 21176210 10.1186/1475-2891-9-69 PMC3019132

[29] Mizutani C , Matsuhashi N , Tomita H , Takahashi T , Suetsugu T , Tajima JY , et al. Predictive value of the prognostic nutritional index in neoadjuvant chemoradiotherapy for rectal cancer. Cancer Diagn Progn. 2022;2:38–48. 35400011 10.21873/cdp.10074 PMC8962848

[30] Kazi M , Gori J , Sasi S , Srivastava N , Khan AM , Mukherjee S , et al. Prognostic nutritional index prior to rectal cancer resection predicts overall survival. Nutr Cancer. 2022;74:3228–3235. 35533003 10.1080/01635581.2022.2072906

[31] Fokas E , Henzel M , Hamm K , Surber G , Kleinert G , Engenhart‐Cabillic R . Multidisciplinary treatment of brain metastases derived from colorectal cancer incorporating stereotactic radiosurgery: analysis of 78 patients. Clin Colorectal Cancer. 2011;10:121–125. 21859565 10.1016/j.clcc.2011.03.009

[32] Kim HJ , Huh JW , Jung TY , Kim IY , Kim HR , Jung S , et al. Clinical outcome with gamma‐knife surgery or surgery for brain metastases from colorectal cancer. J Clin Neurosci. 2013;20:1417–1421. 23910824 10.1016/j.jocn.2012.12.020

[33] Paek SH , Audu PB , Sperling MR , Cho J , Andrews DW . Reevaluation of surgery for the treatment of brain metastases: review of 208 patients with single or multiple brain metastases treated at one institution with modern neurosurgical techniques. Neurosurgery. 2005;56:1021–1034. 15854250

[34] Harland TA , Wang M , Gunaydin D , Fringuello A , Freeman J , Hosokawa PW , et al. Frailty as a predictor of neurosurgical outcomes in brain tumor patients. World Neurosurg. 2020;133:e813–e818. 31605842 10.1016/j.wneu.2019.10.010

[35] Torres‐Perez P , Álvarez‐Satta M , Arrazola M , Egaña L , Moreno‐Valladares M , Villanua J , et al. Frailty is associated with mortality in brain tumor patients. Am J Cancer Res. 2021;11:3294–3303. 34249463 PMC8263656

[36] Dicpinigaitis AJ , Hanft S , Cooper JB , Gandhi CD , Kazim SF , Schmidt MH , et al. Comparative associations of baseline frailty status and age with postoperative mortality and duration of hospital stay following metastatic brain tumor resection. Clin Exp Metastasis. 2022;39:303–310. 35023030 10.1007/s10585-021-10138-3

[37] Heimann M , Schäfer N , Bode C , Borger V , Eichhorn L , Giordano FA , et al. Outcome of elderly patients with surgically treated brain metastases. Front Oncol. 2021;11:713965. 34381733 10.3389/fonc.2021.713965 PMC8350563

